# Prevalence of and factors influencing posttraumatic stress disorder among mothers of children under five in Kabul, Afghanistan, after decades of armed conflicts

**DOI:** 10.1186/1477-7525-6-29

**Published:** 2008-04-23

**Authors:** Kaoruko Seino, Takehito Takano, Taufiq Mashal, Shafiqullah Hemat, Keiko Nakamura

**Affiliations:** 1 International Health, Division of Public Health, Graduate School of Tokyo Medical and Dental University, 1-5-45 Bunkyo, Tokyo 113-8519, Japan; 2 Health Promotion, Division of Public Health, Graduate School of Tokyo Medical and Dental University, 1-5-45 Bunkyo, Tokyo 113-8519, Japan; 3 National Expanded Program on Immunization Office, Ministry of Public Health, Afghanistan; 4 Expanded Program on Immunization section, WHO Country Office, Afghanistan

## Abstract

**Background:**

In the period following wars and other forms of armed conflict, health and quality of life of mothers is a major concern as they have the closest contact with children. The present study was performed to examine the impact of exposure to events related to armed conflicts on post traumatic stress disorder (PTSD) among women raising children, and to identify factors that alleviate the negative consequences of exposure to traumatic events.

**Methods:**

A structured interview survey was conducted in Kabul Province, Afghanistan, in 2006. The subjects were the mothers of children less than 5 years old randomly selected from 1400 households in Kabul Province, Afghanistan. Symptoms of PTSD were assessed according to the criteria of Diagnostic and Statistical Manual of Mental Disorders, Fourth Edition (DSM-IV). Exposure to traumatic events related to armed conflict, experience of hardship with regard to basic needs, resources that the subjects seek for mental health support, and socioeconomic variables were evaluated. Logistic regression analysis was performed to determine the association between PTSD symptoms and predictor variables.

**Results:**

The prevalence rate of PTSD among 1172 women participated in this study was 29.8%. The most prevalent symptom was arousal (74.8%), followed by re-experiencing (54.9%) and avoidance (33.7%). The prevalence rate of PTSD symptoms among subjects who reported having experienced at least one event related to armed conflict (52.7%) was significantly higher than that among those who reported no such experiences (9.6%). Experience of food shortage was independently associated with PTSD. Seeking support for mental health was related to lower prevalence of PTSD symptoms among those who reported no direct experience of events related to armed conflict. However, no such relationship was observed with PTSD symptoms among those who reported having direct experience of events related to armed conflict.

**Conclusion:**

Direct exposure to traumatic events was significantly associated with PTSD symptoms among women raising children. For those who had experienced armed conflict-related events, food security mitigated the occurrence of PTSD symptoms; however, support seeking behavior did not show a significant mitigating influence on PTSD. Means to alleviate the negative influence of exposure to armed conflicts on the quality of life of women should be developed from the viewpoint of quality of mental health support and avoidance of material hardship.

## Background

In the period following wars and other forms of armed conflict, health and quality of life of mothers is a major concern as they have the closest contact with children. The effects of war trauma on the civilian population have more widely reported among women than among men [1,2].

Women in Afghanistan have lived with armed conflict for more than 20 years. Followed by a peaceful life by 1978, Afghanistan experienced an unstable period with armed conflict with factions fighting which continued to the end of 2001 when a military intervention of the international community was made [3]. Although now it is termed 'post-conflict', civilians still face fragile conditions and are still at risk to armed conflicts [4].

While the influence of exposure to traumatic events on the prevalence of stress disorders is widely known [5-7], there has still been relatively little discussion regarding means to restore the quality of life of people who were incapable of fleeing from long periods of conflict.

The objectives of this study were to examine the associations between exposure to armed conflict-related events and prevalence of posttraumatic stress disorder (PTSD) symptoms among women raising children, and to identify factors that may alleviate the negative consequences of exposure to traumatic events.

## Methods

### Subjects

The subjects in the present study were 1400 mothers of children less than 5 years old selected randomly from households in Kabul Province, Afghanistan. Random sampling was conducted based on the list developed with the cooperation and supervision of the Expanded Programme on Immunization Section.

### Procedures

Study teams visited each household to conduct structured interviews in March 2006. Taking into consideration the cultural sensitivity of women in Afghanistan who generally refrain from communicating with males who are not relatives, each study team consisted of one male and one female interviewer, both of whom were trained to administer the interviews. Experience of armed conflict-related events, PTSD symptoms, hardships with regard to basic needs, support seeking resources for mental health, and socioeconomic variables were evaluated.

The protocol of this study was approved by the Ethical Review Board Committee of the Ministry of Public Health, Afghanistan. Informed consent was obtained from all of the subjects.

### Measurements

#### Exposure to armed conflicts-related events

Traumatic events specifically related to armed conflict in Afghanistan over the past decades were assessed. Respondents were asked whether they had experienced shelling or rocket attacks, bomb explosions, murder of family members or relatives, missing or lost of family members, family member becoming disabled because of the conflict, and other events related to armed conflict.

#### Cases of PTSD

PTSD symptoms were assessed according to a scoring algorithm based on the criteria outlined in the Diagnostic and Statistical Manual of Mental Disorders, Fourth Edition, (DSM-IV) criteria for PTSD [8]. The subjects that satisfied all of the three following conditions were defined as subjects with PTSD: reactions on at least 1 of 4 re-experiencing symptoms, at least 3 of 7 avoidance symptoms, and at least 2 of 7 arousal symptoms.

#### Hardships with regard to basic needs

Experience of hardships over the last decade consisted of lack of food, water and shelter was assessed.

#### Resources seeking for mental health support

To examine the coping mechanism for PTSD symptoms, we assessed resources sought by women in Afghanistan for mental health support. The respondents chose multiple resources from among the following listed resources: health facility, religious leader, spiritual healer, friends, reading Quran or praying, holy place, and other.

#### Demographics and socioeconomic variables

Age, number of household member, monthly household income, education, and occupation were examined.

### Analysis

The prevalence of re-experiencing, avoidance, and arousal were calculated as the percentage of the subjects with individual PTSD symptom clusters among the subjects studied in this survey. Prevalence of PTSD was calculated as the percentage of the subjects satisfying all three PTSD symptom cluster criteria among the subjects. The associations between PTSD symptoms and experience of armed-conflict related events, hardships with regard to basic needs, resources sought as mental health support, and socioeconomic variables were examined by logistic regression.

## Results

A total of 1,172 women completed the study (83.7% response rate). Of the respondents, 555 women (47.4%) reported that they experienced at least one traumatic events related to armed conflict. Table 1 shows experienced armed conflict-related events.

**Table 1:** Experiences of traumatic of armed conflict-related events among women in Kabul Province, Afghanistan (n = 1172)

	n	%
Traumatic armed conflict-related events		
Shelling or rocket attacks	213	18.2
Bomb explosion	144	12.3
Disabled family member because of conflict	105	9.5
Murder of family member or relatives	97	8.3
Missing or lost family members	77	6.6
Others	144	12.3
		
Number of armed conflict-related events experienced		
0	617	52.6
1	346	29.5
2	161	13.7
3–7	48	4.1

The prevalence rate of PTSD was 29.8%. The highest symptoms were arousal (74.8%), followed by re-experiencing (54.9%) and avoidance (33.7%).

Demographics and socioeconomic characteristics of the subjects are presented in Table 2. The participants were 21.7 ± 3.6 years old, and among those who had received any education, the mean ± SD educational period was 9.7 ± 3.7 years.

**Table 2:** Demographic and socioeconomic profiles of women in Kabul Province, Afghanistan (n = 1172)

Variables	n	%
Age		
15–19	287	24.5
20–24	666	56.8
25–40	197	16.8
		
Number of family members		
1–5	291	24.8
6–10	746	63.7
>11	135	11.5
		
Monthly household income (US dollars)		
<100	734	62.6
100 ≤ 200	263	22.4
≤200	169	11.4
		
Education		
None	811	71.9
Received	267	22.8
		
Occupation		
Without	1047	89.3
With	118	10.1

Table 3 shows the association between PTSD symptoms and armed conflict-related traumatic events. Experience of conflict-related traumatic events showed a significant association with higher prevalence of PTSD symptoms (p < 0.01).

**Table 3:** Association between PTSD symptoms and traumatic experiences of armed conflict-related events (n = 1172)

				PTSD symptom clusters								
				
	PTSD			Re-experiencing			Avoidance			Arousal		
	OR	95%CI		OR	95%CI		OR	95%CI		OR	95%CI	

Experiences related to armed conflict												
Shelling or rocket attacks	3.37	2.48–4.58	**	15.24	8.88–26.10	**	3.28	2.42–4.46	**	2.54	1.67–3.88	**
Bomb explosions	2.37	1.66–3.39	**	10.15	5.67–18.19	**	2.40	1.69–3.42	**	2.00	1.24–3.22	*
Disabled family member because of conflict	1.31	0.86–2.01		1.73	1.13–2.65	*	1.40	0.93–2.12		1.30	0.79–2.12	
Murder of family members or relatives	4.41	2.86–6.72	**	29.60	9.39–93.43	**	4.82	3.09–7.54	**	1.92	1.09–3.39	*
Missing or loss of family members	3.45	2.15–5.52	**	33.90	8.43–136.8	**	3.78	2.34–6.13	**	2.10	1.09–4.03	*
Others	2.62	1.84–3.74	**	17.45	8.46–35.97	**	2.65	1.86–3.77	**	1.79	1.13–2.83	*
												
Number of armed conflict-related events experienced												
0	1.00			1.00			1.00			1.00		
1	10.02	7.12–14.09	**	49.00	31.24–77.01	**	8.61	6.27–11.84	**	2.91	2.07–4.09	**
2	10.57	7.02–15.90	**	40.10	22.42–71.67	**	10.47	7.05–15.54	**	2.45	1.57–3.50	**
3–7	12.16	6.47–22.83	**	179.30	24.53–1311.2	**	9.67	5.19–18.00	**	4.42	1.72–3.80	**

* p < 0.05, ** p < 0.01

Table 4 shows the association between PTSD and both material deprivation and resources sought for mental health support. Food shortage showed a significant association with PTSD among both subjects who reported having experienced armed conflict-related events and those who reported having not experienced such events. Seeking for mental health support was inversely associated with the prevalence of psychological symptoms among those who reported no experience of armed conflict-related events. There was no statistically significant association with seeking for mental health support and PTSD among those reported having experienced armed conflict-related events. There was no statistically significant association between demographic and socioeconomic variables and PTSD.

**Table 4:** Association between PTSD and material deprivation and support seeking behavior among women who did and did not report having experienced armed conflict-related events

	Experience of events related to armed conflict (n = 555)				No experience of events related to armed conflict (n = 617)			
	PTSD n = 290 (52.7%)				PTSD n = 59 (9.6%)			

	n	OR	95%CI		n	OR	95%CI	

Hardships in basic needs								
Shortage of food	289	2.04	1.45–2.88	**	277	1.86	1.07–3.23	*
Shortage of water	149	1.27	0.86–1.86		193	0.74	0.40–1.36	
Shortage of shelter	285	1.34	0.95–1.88		294	0.59	0.34–1.03	
								
Resources sought for mental health support								
Health facility	492	0.59	0.34–1.02		568	0.27	0.13–0.56	**
Religious leader	339	0.88	0.62–1.24		391	0.48	0.28–0.83	**
Reading Quran or praying	308	0.94	0.67–1.32		390	0.52	0.30–0.90	*
Spiritual Healer	239	0.72	0.50–1.01		352	0.26	0.15–0.48	**
Visiting friends	238	0.73	0.52–1.03		291	0.27	0.14–0.49	**

* p < 0.05, ** p < 0.01

## Discussion

The results of the present study revealed the prevalence of PTSD symptoms and associated factors with PTSD among women raising children in Kabul Province, Afghanistan. PTSD was significantly associated with exposure to traumatic event related to armed conflict and food shortage. Support seeking for mental health resources showed a mitigating influence on armed conflict-related distress among those who reported having no experience of armed conflict-related events.

This study provided evidence from subjects representing a community of Afghanistan that has experienced decades of conflicts with regard to a wide range of determinants of mental well-being. The achieved response rate of 83.7% was attributable to consideration of cultural gender sensitivity in Afghanistan and a full communication with the authorities throughout the research process.

The prevalence rates of PTSD symptoms were relatively high in the present study population in comparison to studies performed in other areas [9-12] both among those who reported having experienced traumatic events related to armed conflict and those who reported having no such experiences. This was considered due to the impact of traumatic events directly related to armed conflict and the exposure to the long-term exposure to the conflicts even among those reporting having no direct experience of armed conflict-related traumatic events.

Material deprivation of food is one of the common traumatic experiences during periods of conflict related to high prevalence of PTSD [13,14]. The independent association between hardship due to lack of food and PTSD symptoms regardless of experience of traumatic events related to armed conflict demonstrated in this study indicates that food security alleviate the negative influence of traumatic events on the occurrence of PTSD symptoms.

Seeking psychosocial support had been identified an effective coping strategy in dealing with traumatic distress [15,16]. Support seeking from health facilities, spiritual healer, and visiting friends tended to show a positive effect on PTSD among subjects who reported direct experience of armed conflict-related traumatic events, although the association was not statistically significant. Considering the significant alleviating influence of seeking support from any resource on PTSD symptoms, these observations indicated the existence of various mental health support resources in Afghanistan society, and their marginal functioning in coping with severe traumatic experiences.

## Conclusion

The present study demonstrated a significant association between direct exposure to traumatic events and PTSD symptoms among women raising children in Afghanistan. Means to alleviate the negative influence on the quality of life of women after exposure to armed conflicts should be developed from the viewpoint of quality of mental health support and prevention of material hardships.

## List of abbreviations

CI: Confidence interval; DSM-IV: Diagnostic and Statistical Manual of Mental Disorders, Fourth Edition; OR: Odds ratio; PTSD: Posttraumatic stress disorder.

## Competing interests

The authors declare that they have no competing interests.

## Authors' contributions

KS conceived the study design and data analysis and drafted the manuscript. TT participated in the design of the study and coordination, TM participated in the study design and conducted a survey, HS took part in data collection and data base preparation and KN participated in the study design, data analysis, and editing the manuscript. All authors read and approved final manuscript.
